# Alpha-Mangostin Reduces Pericellular Fibronectin on Suspended Tumor Cells and Therapeutically, but Not Prophylactically, Suppresses Distant Metastasis

**DOI:** 10.3390/life12091375

**Published:** 2022-09-02

**Authors:** Li-Tzu Huang, Chin-Ho Kuo, Lin Tseng, Yi-Syuan Li, Li-Hsin Cheng, Chin-Yun Cheng, Shane-Rong Sheu, Wen-Tsan Chang, Chien-Chin Chen, Hung-Chi Cheng

**Affiliations:** 1 The Institute of Basic Medical Sciences, College of Medicine, National Cheng Kung University, 1 University Road, Tainan 70101, Taiwan; 2 Division of Hematology-Oncology, Department of Internal Medicine, Ditmanson Medical Foundation Chia-Yi Christian Hospital, Chiayi 600, Taiwan; 3 Department of Cosmetology and Health Care, Min-Hwei Junior College of Health Care Management, Tainan 736, Taiwan; 4 Department of Biochemistry and Molecular Biology, College of Medicine, National Cheng Kung University, 1 University Road, Tainan 70101, Taiwan; 5 The Institute of Biotechnology Research Center, Far East University, Tainan 74448, Taiwan; 6 Department of Pathology, Ditmanson Medical Foundation Chia-Yi Christian Hospital, Chiayi 600, Taiwan; 7 Department of Cosmetic Science, Chia Nan University of Pharmacy and Science, Tainan 717, Taiwan; 8 Department of Biotechnology and Bioindustry Sciences, College of Bioscience and Biotechnology, National Cheng Kung University, Tainan 701, Taiwan

**Keywords:** pericellular fibronectin, suspended tumor cells, tumor metastasis, α-mangostin, mangosteen pericarp extracts, nutraceuticals, therapeutic regime, prophylaxis, AKT phosphorylation, cell survival

## Abstract

Major cancer deaths can be ascribed to distant metastasis to which the assembly of pericellular fibronectin (periFN) on suspended tumor cells (STCs) in the bloodstream that facilitate endothelial attachment can lead. Even though mangosteen pericarps (MP) extracts and the major component α-mangostin (α-MG) exhibit potent cancer chemopreventive properties, whether they can prophylactically and therapeutically be used as dietary nutraceuticals to prevent distant metastasis by suppressing periFN assembly on STCs within the circulation remains obscure. Immunofluorescence staining, MTT assays, flow cytometric assays, immunoblotting, and experimental metastasis mouse models were used to detect the effects of MP extracts or α-MG on periFN on STCs, tumor cell proliferation and apoptosis, the AKT activity, and tumor lung metastasis. The periFN assembly on STCs was significantly diminished upon treatments of STCs with either α-MG or MP extracts in a dose-dependent manner without inhibiting cell proliferation and viability due to increased AKT activity. Pretreatment of STCs with α-MG appeared to suppress tumor lung metastasis and prolong mouse survival rates. Oral gavage with MP extracts could therapeutically, but not prophylactically, prevent lung metastasis of STCs. We concluded that MP extracts or the major component α-MG may therapeutically serve as a potent anti-metastatic nutraceutical.

## 1. Introduction

Tumor metastasis, the major cause of cancer death, is a temporally and spatially multifaceted process that occurs across primary and distant organs. Beginning with the dissemination into the circulation from primary tissues, circulating tumor cells (CTCs) finally colonize specific distant organs to form metastatic cancers [1]. It is, therefore, globally urgent to develop effective therapeutic strategies against often incurable tumor metastasis as current anticancer therapeutic approaches mainly target tumor cells in primary tissues with synthetic cytocidal compounds that have limited efficacies [2,3,4]. Cancerous pericellular fibronectin (periFN) [5,6] has been found to promote colonization and distant metastasis of blood-borne tumor cells [7,8,9]. Indeed, fibronectin (FN) expression critically contributes to tumor malignancy, metastasis, and patients’ poor prognosis [6,10,11,12]. We have reviewed forty years of recent FN-related cancer research and properly justified and rationalized the role of FN in cancer metastasis [10]. Consistently, FN has been reported to be highly expressed in the highly metastatic pancreatic circulating tumor cells (CTCs) [13]. Depletion of endogenously synthesized FN in tumor cells impedes periFN assembly and tumor cells’ lung metastatic potency [14,15]. Moreover, we have previously identified a consensus motif, located in the 13th, 14th, and 15th FN type III repeat fragments that is responsible for binding to dipeptidyl peptidase IV (DPP IV) expressed on the cell surfaces of lung endothelia [9]. The lung metastasis of tumor cells can be blocked by intravenous co-injection of a synthetic peptide that represents this consensus motif and metastatic tumor cells [9]. Therefore, it is practical to target periFN for preventing and suppressing metastasis.

It has been well established that health-promoting properties of plant foods contain phytochemicals that affect biological mechanisms of critical importance to the proper functioning of the human organism [16] but conversely display anticancer therapeutic effectiveness [17], thus being considered a more favorable avenue for cancer prevention and treatment than that of synthetic compounds. Indeed, we have previously found that a well-known phytochemical, pterostilbene, strongly prevents periFN assembly on suspended tumor cells without exerting a cytocidal effect on them and, when orally administered, the lung metastasis of intravenously inoculated tumor cells was greatly reduced [14]. Furthermore, research carried out over the past 20 years has provided evidence suggesting that some regular diets or nutraceuticals are also an essential factor in reducing cancer risk [18]. It would be even better if potentially CTC-targeting edible nutraceutical therapeutics could be given on a daily basis without causing any toxicity prior to and during the presence of CTCs in the circulation for metastatic prophylaxis. 

Many kinds of other plant extracts have exhibited potent cancer chemopreventive properties in the past decade [19,20]. Among them are the extracts of the pericarps of a type of fruit named mangosteen (*Garcinia mangostana*) Linn that grows in the South Asian region [21,22] and displays anticancer properties [22]. It has been reported that α-mangostin (α-MG), the major xanthones secondary metabolites taken from the pericarps of mangosteen, inhibits tumor metastasis by inducing apoptosis of attached tumor cells that could represent tumor cells lodged within either primary or secondary organs [23]. Moreover, xanthone-rich mangosteen liquid taken by healthy human volunteers is considered a nutraceutical and exhibits antioxidant activities to scavenge free radicals that prevent oxidative damage, a risk factor for the development of cancer [24]. Neither does the treatment of peripheral blood monocytes (PBMCs) with α-MG up to 4 µg/mL change the percentages of T cells, B cells, and NK cells nor does it induce either proinflammatory cytokines, i.e., TNF-α and IL-1β or cytokines of adaptive immunity, i.e., IL-2 [25]. However, the effects of xanthone-rich mangosteen extracts and α-MG on cancerous periFN assembly on suspended tumor cells for therapeutic and prophylactic purposes against metastasis have never been examined.

Here, we provide evidence to demonstrate that α-MG is the major component of MP extracts that effectively prevented the assembly of periFN on suspended tumor cell surfaces and suppressed lung metastasis of intravenously inoculated tumor cells. Such inhibitory effects of α-MG and MP extracts were not caused by reduced tumor cell viability. On the contrary, treatment of α-MG promoted the survival signal AKT phosphorylation in suspended tumor cells, explaining why it did not affect tumor cell viability. Expectedly, oral administration of MP extracts therapeutically prevented intravenously inoculated tumor cells from metastasizing to the lungs. However, pre-orally giving MP extracts before tumor cell injection resulted in more severe lung metastasis, suggesting that MP extracts or α-MG serves as a cancer therapeutic, after the presence of CTCs in the circulation, but not as a prophylactic, before their presence, regime against tumor metastasis.

## 2. Materials and Methods

### 2.1. Cell Lines

Both Lewis lung carcinoma (LLC) cell line (ATCC: CRL-1642, Richmond, VA, USA), purchased from the American Type Culture Collection, and mouse mammary carcinoma cell lines (4T1) cell line, a generous gift from Dr. F. Miller from Karmanos Cancer Institute, Wayne State University, Detroit, MI, were cultured in Dulbecco’s modified Eagle’s medium (DMEM) containing 10% fetal bovine serum (FBS) (Invitrogen, Karlsruhe, Germany) and supplemented with 2 mM L-glutamine for LLC cells and additional 1 mM sodium pyruvate (Sigma G6013, St. Louis, MO, USA) for 4T1 cells. Tissue culture plasticware was purchased either from Wuxi NEST. Biotechnology Co., Ltd. (Wuxi, China) or from BD Falcon (Franklin Lakes, NJ, USA). To prepare tumor cells in suspension, sub-confluent cells grown in and attached to culture dishes were trypsinized with 0.25% trypsin and 0.02% EDTA in phosphate-buffered saline (PBS) at 37 °C for 15–30 s, washed once in DMEM containing 10% FBS, and then subjected to end-over-end (EoE) rotation for 2 h at 37 °C in 1.5-mL centrifuge tubes in 20% FBS-containing DMEM with a cell density of 1 × 10^6^ cells/mL.

### 2.2. Materials

Polyclonal antibody (pAb) against FN (α-FN pAb) and EDTA were purchased from Sigma-Aldrich, Inc. (St. Louis, MO, USA). Anti-pAKT pAb was purchased from Abcam (Cambridge, MA, USA). Anti-AKT rbt pAb was purchased from ABclonal Technology (Woburn, MA, USA). Anti-GAPDH rbt pAb was purchased from Elabscience Biotechnology Inc (Houston, TX, USA). Goat anti-rabbit (GαR) IgG-Alexa 488 (A11008) was purchased from Invitrogen (Waltham, MA, USA). Control rabbit non-immune IgG was purchased from Jackson Immunoresearch Laboratories, INC (West Grove, PA, USA). MTT powder was purchased from VWR (Radnor, PA, USA). Bovine serum albumin (BSA) and CyECL luminescence reagents for western blotting were purchased from Cyrusbioscience (Taipei, Taiwan). Propidium iodide (PI) was purchased from Invitrogen (Waltham, MA, USA). Annexin V-FAM apoptosis detection reagent was purchased from LEADGENE (Tainan, Taiwan). Immobilon-P Poly vinylidene fluoride (PVDF) Membrane (IPVH00010) was purchased from Sigma-Aldrich, Merck Millipore (Darmstadt, Germany).

### 2.3. Extraction of Mangosteen Pericarp

*Garcinia mangostana* was obtained from a traditional market in Thailand. After removing the pulp, the mangosteen pericarp (MP) was dried and stored in a sealed container. MP was first crushed into MP powder, followed by filtration with 20 mesh sieve sizes before being subjected to low-temperature extraction. The procedure for low-temperature extraction was to mix 300 g MP powder and 3 kg 95% ethanol in a 5 L glass extraction container harboring a high-speed homogenizer to generate strong shear force for breaking down cell walls. A simultaneously turned-on ultrasound machine was placed under the glass container at 40 kHz to promote the extraction efficiency for 1 h at 40 °C. A final 2.65 kg of MP extracts was collected after being subjected to filtration with a Büchner funnel. After vacuum concentration to remove ethanol, the MP extracts were mixed with 0.8 kg dextrin excipient before being subjected to freeze-drying. The concentrated materials were ground, and 1.06 kg of MP powder was produced.

### 2.4. Immunofluorescence (IF) Staining for periFN on Suspended Tumor Cells

Suspended LLC and 4T1 cells were treated with control vehicle (DMSO), MP extracts, or xanthone-derived phytochemicals at various concentrations and conditions as indicated wherever appropriate before being subjected to IF staining for periFN as previously described [15]. Briefly, cells grown in culture dishes to sub-confluence were trypsinized with 0.25% trypsin, 0.02% EDTA in phosphate-buffered saline (PBS) at 37 °C, washed once in DMEM containing 10% FBS, and subjected to EoE suspension culture for 2 h at 37 °C in 1.5-mL centrifuge tubes in 20% FBS-containing DMEM with a cell density of 1 × 10^6^ cells/mL. Tumor cells were then washed once with PBS prior to the 1 h incubation at 4 °C with α-FN pAb and 1 h incubation at 4 °C with secondary GαR IgG-Alexa 488, both in PBS containing 1% BSA. After that, cells were washed once with PBS and fixed with 1% paraformaldehyde (PFA) in PBS at room temperature for 10 min. Images were photographed for fluorescence under an Olympus IX71 fluorescence microscope (Shinjuku, Tokyo, Japan). The fluorescence intensity for each cell in images was calculated with Image J software. BD FACSCalibur™ flow cytometer was used to acquire data resulting from 3000~10,000 cells. The cell sizes were monitored by forward scatter (FSC), and FN^+^ cells detected in the FL1 channel were calculated in dot plots as compared with cells stained with non-immune IgG.

### 2.5. MTT Assay

LLC and 4T1 cells were subjected to EoE suspension culture in DMEM containing 0.5% BSA and α-MG (30, 50, 60 mM or control DMSO) for 30 min at 37 °C, followed by the addition of FBS to make a final 20% concentration prior to a 1.5-h cell recovery at 37 °C. Cells were centrifuged for 1000 rpm for 3 min at room temperature (RT) to remove α-MG before being seeded into 48-well microplates at 2500 cells/well in the presence of DMEM containing 10% FBS. The cell proliferation rates were measured at 24, 48, and 72 h with MTT assays according to manufacturer′s instructions [14].

### 2.6. Apoptosis Assay

Quantifications of tumor cell apoptosis were performed employing PI/Annexin V-FAM double fluorescence staining methodology as previously described [14,26]. Briefly, tumor cells treated with DMSO or α-MG were grown in EoE suspension and cultured for 24 h at 37 °C prior to PI/Annexin V-FAM double fluorescence staining. The stained tumor cells were subjected to flow cytometry and FACS analysis. A total of 3000 cells were acquired, and the cell percentages in each quadrant were calculated after gating according to the results of non-stained cells.

### 2.7. Immunoblotting

LLC cells treated with DMSO or α-MG were lysed to make cell lysates and subjected to SDS-PAGE electrophoresis and western immunoblotting (IB) on PVDF membranes according to previously described protocols [26]. The PVDF membranes were treated with CyECL reagents, and chemiluminescence IB images were taken from UVP BioSpectrum**^®^**AC imaging system (Analytik Jena US, Upland, CA, USA) according to the manufacturer’s instructions. Internal loading controls were performed with Coomassie Blue staining on the same blotted PVDF membranes.

### 2.8. Experimental Tumor Metastasis Animal Models

All animal experiment protocols in this study were performed according to the Guide for Care and Use of Laboratory Animals at National Cheng Kung University (NCKU) and approved by NCKU Internal Laboratory Animal Care and Use Committee at NCKU Laboratory Animal Center. C57BL6 and BALB/c mice were acquired from NCKU Laboratory Animal Center and housed in groups of five mice per cage. For lung metastasis of LLC, cells were pretreated with DMSO or α-MG before intravenous inoculation. A total of 5 × 10^5^ LLC cells were similarly treated with DMSO or α-MG as previously described [14] before tail vein injection into C57/BL6 mice (5 mice in each group). After 30 days, mice were sacrificed with CO_2_ euthanasia and mouse lungs were freshly taken or subjected to formalin fixation for lung photography, tumor nodule counting, and H&E histological staining. For evaluating the therapeutic and prophylactic effects of MP extracts as a nutraceutical beverage on lung metastasis of circulating LLC or 4T1 cells, we divided C57BL6 or BALB/c mice into four or three groups (five mice in each group), respectively, in which mice orally received either drinking water (MQ) or MP extracts thrice a week for one month before (Before) or after (After) tail vein injection of 5 × 10^5^ LLC or 4T1 cells. For LLC cells, there was an additional group where mice drank MP extracts once per day for two days before intravenously receiving tumor cells and continued to be fed with MP extracts once per day only for two more days (Before + After). Afterward, mice were stopped from drinking MP extracts till scarification on the 30th day. Upon scarification after one month, mice were subjected to CO_2_ euthanasia and mouse lungs were subjected to formalin fixation for lung photography, tumor nodule counting, and H&E histological staining. 

### 2.9. Statistical Analysis

GraphPad Prim6 was used to perform statistical analyses. The comparisons of paired data were analyzed with Student’s t-test. For the means of results from two or more independent groups, one-way ANOVA was employed. Two-way ANOVA analyzed variance and test differences in the effects of independent variables on a dependent variable such as tumor cell proliferation. All experiments were at least independently performed in biological triplicate, and the results are shown as mean ± S.D. The statistical differences were represented by the *p* value in which *p* < 0.05 (*), *p* < 0.01 (**), *p* < 0.001 (***), *p* < 0.0001 (****) were considered as different levels of significance (the lower p value, the higher significance).

## 3. Results

### 3.1. Extracts of Mangosteen Pericarps Suppress periFN Assembly on Tumor Cells in Suspension

**Figure 1:**
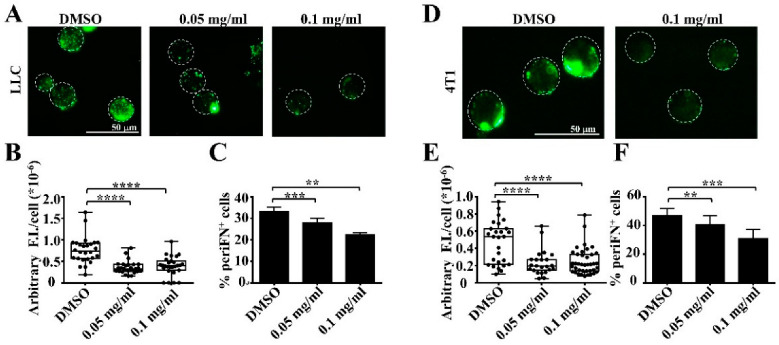
Extracts of mangosteen pericarps dose-dependently suppress periFN assembly on suspended tumor cells. (**A**) Fluorescence immunostaining for periFN that was assembled on suspended LLC tumor cells in the absence or presence of various concentrations, as indicated, of extracts of mangosteen pericarps (MP extracts). (**B**) Quantification of the arbitrary fluorescence intensity (F.I.)/cell for the images in (**A**). (**C**) Quantification of the immunostained cells in (**A**) with FACS analysis. (**D**–**F**) Similar fluorescence staining and quantifications for 4T1 suspended tumor cells as performed in (**A**–**C**) for LLC cells. **, *p* < 0.01; ***, *p* < 0.001; ****, *p* < 0.0001.

To examine whether extracts of mangosteen pericarps (MP extracts) exert an inhibitory effect on periFN assembly on suspended tumor cells, we pretreated LLC lung tumor cells in suspension with MP extracts and subjected cells to 2-h recovery in culture media containing 20% FBS, followed by immunofluorescence staining with α-FN pAb. We found that periFN assembly on suspended LLC cells was dose-dependently suppressed by MP extracts (Figure 1A–C). Next, we asked whether the suppressive effect of MP extracts on periFN assembly was limited to lung tumor cells. We pretreated other well-known mouse mammary adenocarcinoma 4T1 cells with MP extracts and similarly demonstrated a dose-dependent suppression of periFN assembly on suspended 4T1 cell surfaces (Figure 1D–F). Altogether, our results suggest that the inhibitory effect of periFN assembly on suspended tumor cells is not in a tumor-type-specific manner. 

### 3.2. Alpha-MG, but Not Xanthone, in MP Extracts Suppresses periFN Assembly on Suspended Tumor Cells

**Figure 2:**
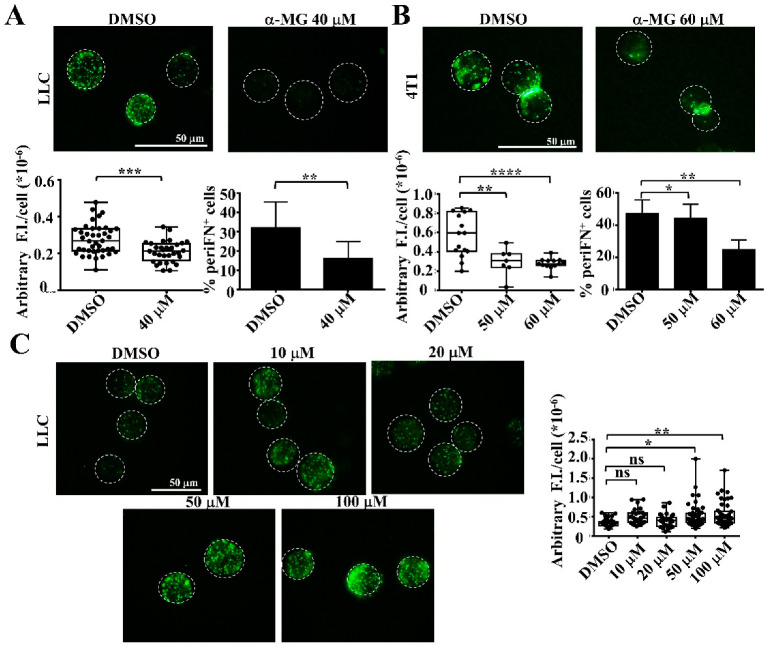
α-MG suppresses periFN assembly on suspended tumor cells. (**A**) Fluorescence immunostaining for periFN that was assembled on suspended LLC tumor cells in the absence or presence of 40 μM of α-MG (upper panel). The quantifications of the arbitrary fluorescence intensity (F.I.)/cell for the images (lower left panel) and FACS analysis for the immunostained cells (lower right panel). (**B**) Similar fluorescence immunostaining and quantifications for 4T1 suspended tumor cells as performed in (**A**–**C**) for LLC cells. (**C**) Fluorescence immunostaining for periFN that was assembled on suspended LLC tumor cells in the absence or presence of various concentrations, as indicated, of Xanthone and the quantifications of the arbitrary fluorescence intensity (F.I.)/cell for the images. *, *p* < 0.05; **, *p* < 0.01; ***, *p* < 0.001; ****, *p* < 0.0001; ns, no significance.

Because α-MG has been known as the major component in MP extracts, we treated suspended LLC (Figure 2A) or 4T1 (Figure 2B) cells with various concentrations of α-MG, instead of MP extracts, and found that periFN assembly on both suspended tumor cell lines was significantly inhibited. Next, xanthone was used as a control component in MP extracts and showed that there was no difference in periFN assembly on LLC cells at lower concentrations (Figure 2C: 10 and 20 μM) of xanthone, and the periFN assembly was even slightly increased at higher concentrations (Figure 2C: 50 and 100 μM). These results indicate that α-MG is the major effective component in suppressing periFN assembly on tumor cell surfaces.

### 3.3. Alpha-MG Treatment Does Not Affect Tumor Cell Viability in Suspension Due to Increased AKT Activity

**Figure 3:**
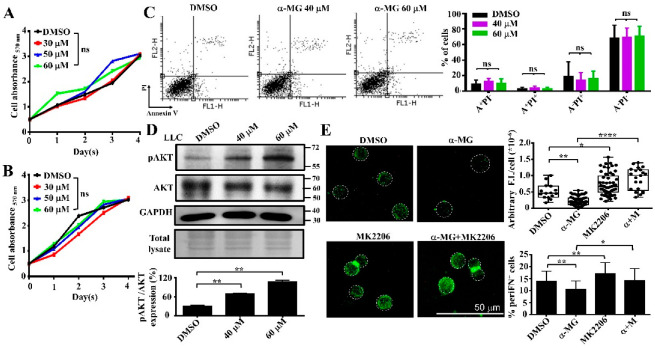
Treatment of suspended tumor cells with α-MG does not affect cell viability but increases AKT activity. (**A**) Cell proliferation rates of LLC cells treated with various concentrations of α-MG when tumor cells were grown in suspension for two hours before being seeded in 48-well microplates and the cell proliferation was detected with MTT assays. (**B**) Cell proliferation rates of 4T1 cells similarly treated as in (**A**). (**C**) Apoptosis rates for LLC cells (% LLC cell death) treated with vehicle (DMSO), 40, or 60 μM of α-MG in suspension for 24 h and stained with PI/Annexin V in apoptosis assays (**A**: annexin V, PI: propidium iodide). Left panel: representative flow cytometry dot plots, Right panel: quantification of three independent experiments. (**D**) Phosphorylation of AKT (pAKT) in LLC cells similarly treated with DMSO or α-MG as in (**A**) and immediately subjected to cell lysing for western blotting without being seeded in microplates (two upper panels: pAKT and total AKT, third panel: GAPDH as a loading control). Coomassie blue staining of the blotted membrane for pAKT detection was used as an alternative loading control (fourth panel). Quantification of the pAKT/total AKT (%) was measured with the densitometry tool in software Image J (lower panel). (**E**) Fluorescence immunostaining for periFN that was assembled on suspended LLC tumor cells treated with DMSO, α-MG (40 μM), MK2206 (AKT inhibitor: 0.2 μM), or α-MG+MK2206, the quantifications of the arbitrary fluorescence intensity (F.I.)/cell for the images. *, *p* < 0.05; **, *p* < 0.01; ****, *p* < 0.0001; ns, no significance.

**Figure 4:**
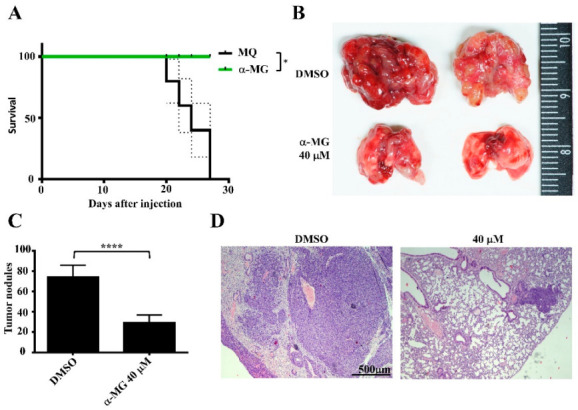
Lung metastasis of intravenously inoculated tumor cells pretreated with α-MG is effectively inhibited. (**A**–**D**) LLC cells were pretreated with culture media containing vehicle DMSO or 40 μM of α-MG prior to tail vein injection. Thirty days later, mouse survival (**A**) was measured, and lungs were taken for imaging (**B**), nodule counting (**C**), and H&E staining (**D**) upon mouse sacrifices. *, *p* < 0.05; ****, *p* < 0.0001.

The inhibitory effect of α-MG on periFN assembly on suspended tumor cells could be due to reduced cell viability that disables the suspended tumor cells from assembling periFN. In order to test this possibility, we first treated suspended LLC (Figure 3A) and 4T1 (Figure 3B) cells with α-MG for 2 h before seeding cells in 48-well microplates for cell proliferation assays. We showed that the viabilities and proliferative activities of suspended cells treated with all concentrations of α-MG were not changed as compared with cells treated with DMSO vehicle (Figure 3A,B). Next, we tested whether α-MG triggers *necrosis and/or* apoptosis of tumor cells in suspension for 24 h. Employing PI/Annexin V apoptosis assays, we showed that no *significant cell death* (neither necrosis nor apoptosis) of LLC cells was induced at either 40 or 60 μM α-MG (Figure 3C), altogether, suggesting that the inhibitory effect of α-MG on periFN assembly on suspended tumor cells is not due to reduced viability and induced *cell* death. These results were highly reminiscent of those of another study which showed that pterostilbene increases AKT activity in suspended tumor cells, leading to cell survival signaling via the AKT/ERK axis [14]. To examine such phenomenon is also true for the unchanged viability with α-MG treatment, we treated suspended LLC cells with α-MG for 2 h and subjected cell lysates to western blotting for AKT phosphorylation and *total AKT*. We found that AKT phosphorylation was induced by α-MG in a dose-dependent manner (Figure 3D), rationalizing why the viability of suspended was not affected by α-MG, which is reportedly potent in triggering apoptosis of attached tumor cells [27]. Finally, we combined α-MG and AKT inhibitor MK2206 for treating tumor cells and found that the suppressed periFN assembly was restored (Figure 3E), suggesting that α-MG suppresses periFN assembly on suspended tumor cells via enhanced AKT activity.

### 3.4. Pretreatment of Suspended Tumor Cells with α-MG Prior to Intravenous Inoculation Reduces Lung Metastasis

The periFN assembly on suspended tumor cell surfaces has unequivocally been deemed as a required factor in promoting endothelial adhesion, lung colonization, and eventual metastasis [14,15]. We asked whether α-MG-suppressed periFN causes in vivo lung metastasis of tumor cells. We showed that pretreating suspended LLC cells with α-MG resulted in prolonged mouse survival (Figure 4A) and reduced lung metastasis (Figure 4B). Consistently, the tumor nodule numbers in mouse lungs were significantly decreased (Figure 4C,D).

### 3.5. Oral Gavage with MP Extracts Therapeutically, but Not Prophylactically, Suppresses Lung Metastasis of Tumor Cells

**Figure 5:**
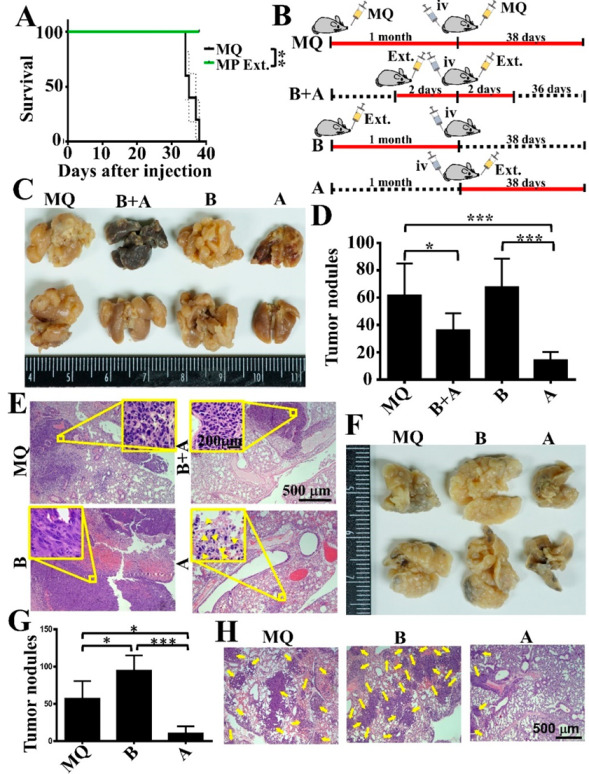
Orally administered extracts of mangosteen pericarps therapeutically, but not prophylactically, suppress lung metastasis of intravenously inoculated tumor cells. (**A**) Mice were orally given drinking water (MQ) or extracts of mangosteen pericarps thrice a week for 6 weeks right after tail vein injection of LLC cells, and the survivals were recorded, and survival curves were plotted. (**B**) Illustration for the therapeutic and prophylactic schemes of oral gavage with extracts of mangosteen pericarps (Ext.) against LLC metastasis in the lungs. Note: Solid lines in red indicate oral gavage with MQ or Ext. Broken lines in black indicate the time periods without any oral gavage. (**C**–**E**) Mouse lungs were taken for imaging (**C**), nodule counting (**D**), and H&E staining (**E**) upon mouse sacrifices. Note: the magnitudes of the insets are 10 times that of the images in (**E**) to demonstrate the details of the tumor tissues. Arrows indicate fragmented chromosomes in apoptotic tumor cells. (**F**–**H**) Similar schemes as in (**B**) for 4T1 metastasis in the lungs to evaluate therapeutic and prophylactic effects of extracts of mangosteen pericarps. Mouse lungs were taken for imaging (**F**), nodule counting (**G**), and H&E staining (**H**) upon mouse sacrifices. Note: arrows in (**H**) indicate lesions of tumor nodule tissues. *, *p* < 0.05; **, *p* < 0.01; ***, *p* < 0.001.

Now that we showed α-MG is the major component in MP extracts responsible for the suppression of periFN-promoted tumor metastasis, we were next interested in knowing whether MP extracts could serve as a nutraceutical to exert not only therapeutic but also prophylactic effects on metastatic suppression. To begin with, we simply performed animal survival assays and tested the effect of oral gavage with drinking water (MQ) or MP extracts thrice a week right after intravenous inoculation of LLC cells. Mice that orally received MP extracts had significantly prolonged survival compared with mice that received MQ (Figure 5A), clearly indicative of a therapeutic effect of MP extracts as a nutraceutical regime. We further designed and evaluated the therapeutic and prophylactic effects of orally administered MP extracts on lung metastasis of tumor cells (Figure 5B). Expectedly, compared with mice receiving MQ, oral gavage with MP extracts thrice a week right after intravenous LLC cell inoculation drastically suppressed lung metastasis, nodule numbers in the mouse lungs, and metastatic tumor lesions (Figure 5C–E: MQ and After (A)), confirming that MP extracts can serve as a nutraceutical to therapeutically treat tumor cells in the circulation via controlling periFN on suspended tumor cells and in distant tissues most likely via exerting cell apoptosis and necrosis effects (Figure 5A,E). Unfortunately, when MP extracts were orally given to naïve mice thrice a week for one month and the MP extract oral gavage was stopped right before tumor inoculation, the MP extracts did not only lose the anti-metastatic potency but, conversely, slightly increased lung metastasis (Figure 5C–E: MQ and Before (B)), suggesting that the MP extracts are not suitable to serve as a prophylactic regime before the presence of tumor cells in the circulation to prevent lung metastasis. Moreover, when mice only orally received MP extracts for two days before and continuously for two days after intravenous LLC cell inoculation, despite the fact that MP extracts sustained anti-metastatic effect (Figure 5C–E: MQ and Before + After (B + A)), but the inhibitory potency was significantly reduced (Figure 5C–E: B + A and A), reconfirming that MP extracts are absolutely inappropriate to be given orally before the presence of tumor cells in the circulation. When we performed similar experimental metastasis animal models for 4T1 tumor metastasis in the lungs, we yielded the same conclusion as for LLC cells (Figure 5F–H).

## 4. Discussion

Many pharmacological properties of α-MG, the most abundant xanthone derived from the mangosteen pericarps, have been reported, including inhibitory activities on oxidative stress, pathogen infections, diabetes, and carcinogenesis [28]. It also serves as a nutraceutical to be neuroprotective, hepatoprotective, and cardioprotective [17]. Among these, anticancer effects are the most promising property for therapeutic and prophylactic purposes [29]. Much research on α-MG suggests that it can prevent distant metastasis by inducing cell apoptosis when primary tumor cells are pretreated [24,28,30]. To our best knowledge, we are the first to identify α-MG as an inhibitor of cancer metastasis by targeting and suppressing periFN assembly on suspended tumor cells in the circulation without triggering cell apoptosis (Figure 1, Figure 2 and Figure 3), which was rationalized by the increased AKT phosphorylation (Figure 3D) as a survival signal [30]. These results also reason why and how α-MG effectively suppresses metastasis of intravenously injected colorectal cancer cells [31]. Interestingly, the effect of α-MG on periFN suppression on suspended tumor cells without causing apoptosis is coincident with that of pterostilbene, implicating the likelihood that both phytochemicals are involved in the same or similar AKT-suppressed pathway, leading to disassembly of periFN on suspended tumor cells.

The way α-MG affects attached tumor cells by triggering cell apoptosis [23] and on suspended tumor cells by suppressing their periFN assembly (Figure 2) and activating AKT (Figure 3D) is highly reminiscent of the functions of pterostilbene [14], implying that these two phytochemicals target molecules that are involved in the same pathway within adherent tumor cells to cause apoptosis and within suspended cells to disassemble periFN and resist anoikis. AKT apparently plays a central role in distinctly regulating the functions of adherent and suspended tumor cells. It has been well known that AKT activity is an essential component in the pathway sustaining the survival of solid tumor cells [32]. In congruence with this concept, both α-MG and pterostilbene have continuously been reported to downregulate the PI3K/AKT pathway, accounting for apoptotic induction [14,23,33,34,35,36]. Contrarily, both α-MG (Figure 3D) and pterostilbene [14] increase phosphorylation of AKT and suppress periFN assembly on suspended tumor cells, legitimately reasoning why tumor cells treated with these two compounds in suspension are resistant to anoikis, a special type of cell apoptosis that occurs due to AKT inactivation and a loss of cell attachment [37]. While there is ample research elaborating the mechanism underlying how fibrillary FN matrices on adherent cells are assembled [6], the signal pathway directing anoikis and periFN assembly on suspended tumor cells is less clear. We have previously disclosed that periFN assembly on blood-borne tumor cells is regulated by PKCε [15]. PKCε is a multifunctional protein that regulates cancer development and progression [38] and EMT-related cell apoptosis and anoikis [39,40]. Now that both α-MG and pterostilbene suppress periFN assembly on suspended cells, the reason treatment with α-MG or pterostilbene renders suspended cells more resistant to anoikis is highly likely due to the inhibition of PKC**ε** signaling. Indeed, the blockade of PKC**ε** ameliorates anoikis of tumor cells grown in suspension [41]. It has been reported that AKT mediates tumor cell anoikis resistance [42], rationalizing the increased AKT phosphorylation and survival of tumor cells treated with α-MG or pterostilbene in suspension (Figure 3).

After we demonstrated the therapeutic effect of α-MG against cancer metastasis (Figure 4 and Figure 5A), we further hoped to employ α-MG as a prophylactic nutraceutical on a daily basis. However, we, unfortunately, found that daily oral gavage with α-MG before intravenous injection of tumor cells unexpectedly exacerbated the lung metastasis of LLC cells (Figure 5C–H). Pre-injured vasculature caused directly by α-MG uptake or indirectly by α-MG-triggered autoimmunity is a major possibility that could have resulted in the reduced integrity of blood vessels and enhanced tumor cell extravasation within the circulation. Although α-MG is considered a nutraceutical displaying antioxidant effects [24] and, when PBMCs are treated with α-MG, the percentages of various blood cell types, including T, B, and NK cells, and secretions of several major proinflammatory and adaptive immune cytokines are not significantly changed [25], in vivo oral administration of α-MG stimulates TNF-α secretion in primary human blood monocyte-derived macrophages [43] and increases serum levels of IL-1 and complement components [44], which may directly lead to direct vascular injury and blood vessel integrity devastation [45,46,47,48]. 

Alternatively, cumulative evidence suggests that vascular injury may indirectly be ascribed to a dysregulated autoimmune response [49]. Ample evidence has indicated the competence of natural killer (NK) cells in killing lung endothelial cells and posing pulmonary vascular hyperpermeability and injury [50,51,52,53]. One of major NK cell activators is stimulator of interferon genes (STING), an endoplasmic reticulum (ER) dimeric adapter transmembrane protein expressed in various endothelial and epithelial cells and hematopoietic cells such as macrophages, DC, and T cells, lying in the cGAS-STING-TBK1 signaling pathway. STING acts as a master regulator of type I interferon (IFN) production and the innate immune system [54], inducing the secretion of the chemokines CCL5 and CXCL10 to facilitate the activation of NK cells [55]. Importantly, α-MG has been identified as an agonist of STING and employed to serve as a STING-activating vaccine adjuvant [56,57]. Conceptually, α-MG may target the STING pathway in endothelial, epithelial, or immune cells for circulating CCL5/CXCL10-mediated NK activation, leading to pre-injured lung vasculature and enhanced vessel permeability, which facilitates the entering of circulating tumor cells into the lung parenchyma and metastatic development. Such a hypothesis is worthy of further investigation in the future.

α-MG could only therapeutically suppress distant metastasis via disassembling periFN on suspended tumor cells, and it prophylactically increased the lung metastasis when pre-administered to mice before tail vein injection of tumor cells (Figure 5). Nevertheless, tumor cells in the circulation are derived from primary tissues where they attach to the extracellular matrices within microenvironments [1]. Therefore, pretreating tumor cells with α-MG in the primary sites remains useful, as it can still exert potent apoptotic, anti-migratory, and anti-invasive effects on these adherent cells and prevent the intravasation of tumor cells from becoming CTCs [29] and eventually distant metastasis [23]. To avoid the potential direct or indirect vascular injury caused by α-MG as a daily consumed nutraceutical and increased lung metastasis once CTCs emerge in the circulation, medicinal plants, fruits, or vegetables known for vascular protection [58] may be taken as additional supplements hopefully for prophylactic anti-metastatic purposes. Vascular endothelial dysfunction can be manifested by several characteristics, including an imbalance of vasodilation and vasoconstriction, deficiency of nitric oxide (NO) bioavailability and elevated reactive oxygen species (ROS), and proinflammatory factors, ending up with increased vascular permeability, which reportedly can be reversed by several phytochemical compounds, e.g., curcumin, resveratrol, cyanidin-3-glucoside, berberine, epigallocatechin-3-gallate, and quercetin, contained in the above-described natural plant products [58,59]. According to a recent review article, resveratrol, among these phytochemicals, is of particular interest and promising for vascular protective purposes through multiple molecular mechanisms [60]. Together with vascular protecting nutraceuticals, α-MG may ideally serve as a prophylactic and therapeutic plant medicine against tumor formation, dissemination through the circulation, and metastatic development.

## 5. Conclusions

**Figure 6:**
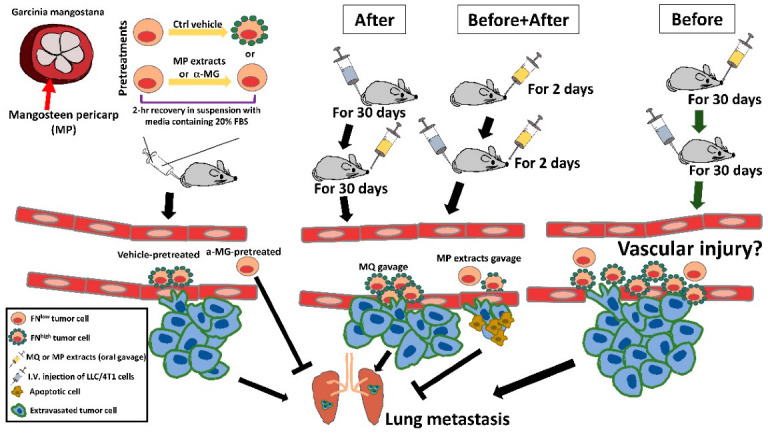
Schematic illustration of the inhibitory effects of α-MG or MP extracts, when incubated with suspended tumor cells or orally administered to mice, therapeutically, but not prophylactically, on lung metastasis of intravenously inoculated tumor cells.

In summary, our results suggest that MP extracts or the major component α-MG in MP extracts, in spite of being unsuitable for prophylaxis, may therapeutically serve as a potent anti-metastatic nutraceutical for suppressing lung metastasis of blood-borne tumor cells (Figure 6).
